# Ethnic differences in the incidence of pterygium in a multi-ethnic Asian population: the Singapore Epidemiology of Eye Diseases Study

**DOI:** 10.1038/s41598-020-79920-9

**Published:** 2021-01-12

**Authors:** Xiao Ling Fang, Crystal Chun Yuen Chong, Sahil Thakur, Zhi Da Soh, Zhen Ling Teo, Shivani Majithia, Zhi Wei Lim, Tyler Hyungtaek Rim, Charumathi Sabanayagam, Tien Yin Wong, Ching-Yu Cheng, Yih-Chung Tham

**Affiliations:** 1 Ocular Epidemiology, Singapore Eye Research Institute, Singapore National Eye Centre, The Academia, 20 College Road, Discovery Tower Level 6, Singapore, 169856 Singapore; 2 Department of Ophthalmology, Shanghai Eye Diseases Prevention &Treatment Center/ Shanghai Eye Hospital, Shanghai, China; 3 Ophthalmology and Visual Sciences Academic Clinical Program (Eye ACP), Duke-NUS Medical School, Singapore, Singapore; 4 Department of Ophthalmology, Yong Loo Lin School of Medicine, National University of Singapore, Singapore, Singapore

**Keywords:** Corneal diseases, Risk factors

## Abstract

We evaluated the 6-year incidence and risk factors of pterygium in a multi-ethnic Asian population. Participants who attended the baseline visit of the Singapore Epidemiology of Eye Diseases Study (year 2004–2011) and returned six years later, were included in this study. Pterygium was diagnosed based on anterior segment photographs. Incident pterygium was defined as presence of pterygium at 6-year follow-up in either eye, among individuals without pterygium at baseline. Multivariable logistic regression models were used to determine factors associated with incident pterygium, adjusting for baseline age, gender, ethnicity, body mass index, occupation type, educational level, income status, smoking, alcohol consumption, presence of hypertension, diabetes and hyperlipidemia. The overall age-adjusted 6-year incidence of pterygium was 1.2% (95% confidence interval [CI] 1.0–1.6%); with Chinese (1.9%; 95% CI 1.4%-2.5%) having the highest incidence rate followed by Malays (1.4%; 95% CI 0.9%-2.1%) and Indians (0.3%; 95% CI 0.3–0.7%). In multivariable analysis, Chinese (compared with Indians; odds ratio [OR] = 4.21; 95% CI 2.12–9.35) and Malays (OR 3.22; 95% CI 1.52–7.45), male (OR 2.13; 95% CI 1.26–3.63), outdoor occupation (OR 2.33; 95% CI 1.16–4.38), and smoking (OR 0.41; 95% CI 0.16–0.87) were significantly associated with incident pterygium. Findings from this multi-ethnic Asian population provide useful information in identifying at-risk individuals for pterygium.

## Introduction

Pterygium, a common ophthalmic condition among elderly, is characterized by an overgrowth of bulbar conjunctiva, which may encroach the central cornea at advanced stages, thereby inducing significant astigmatism and causing visual impairment^1,2^.

Previous population studies showed that the prevalence of pterygium varied greatly between 1.3% and 39.5% across ethnicity and geographic regions, with Chinese ethnicity living in rural China reporting a higher prevalence in pterygium (Supplemental Table 1). However, given that these previous studies were conducted in different countries/ regions with different ultraviolet light exposure level, direct comparisons between different ethnic groups was difficult. The Barbados Eye Study reported that in the west indies, individuals with darker skin have a lower incidence of pterygium than those with lighter skin^3^, suggesting that skin pigmentation may influence the risk of pterygium.

Furthermore, information on the incidence of pterygium is scarce. To the best of our knowledge, only four studies(Supplemental Table 2) have documented the incidence of pterygium: the Barbados Eye Study^3^, the Beijing Eye Study^4^, the Yunnan Minority Eye Study^5^ and the Korean cohort study^6^. However, these past studies mainly focused on a single ethnic group in their evaluations. Inter-ethnic comparison on pterygium incidence has not yet been documented among Asians. Given the large variation among study results in pterygium prevalence across ethnicities difference reported in previous study (Supplemental Table 1) and lack of inter-ethnic comparison on pterygium incidence, we hypothesize that the cumulative incidence and risk factor profiles may be different across the three ethnic groups in Singapore.

Singapore has three major ethnic groups: Chinese, Malay, and Indian origin, which are also the three major ethnic groups in Asia. This provides a unique opportunity to examine the possible ethnic differences in pterygium incidence in Asians. Hence, the aim of this study was to compare the incidence and risk factors of pterygium in a multi-ethnic Asian population in Singapore.

## Methods

### Study population

Participants were recruited from the Singapore Epidemiology of Eye Diseases (SEED) study, which is a population-based cohort study comprising of adults aged 40 years and above from the 3 major ethnic groups in Singapore: Malay, Indian, and Chinese. Methodology and details of the SEED study have been previously reported^7–12^. In brief, an age-stratified random sampling method was used to select Malays, Indians and Chinese from the southwestern part of Singapore. Baseline examinations were conducted between 2004–2011 and included 3,280 Malays (2004–2006, response rate 78.7%)^7^, 3,400 Indians (2007–2009, response rate 75.6%)^8^, and 3,353 Chinese (2009–2011, response rate 72.8%)^9^. The 6-year follow-up examinations were conducted between 2010–2017 and included 1,901 Malays (2010 to 2014, response rate 72.1%)^10^, 2,200 Indians (2013 to 2015, response rate 75.5%)^11^, and 2,661 Chinese (2015–2017 response rate 87.7%)^12^.

All study procedures adhered to the principles of the Declaration of Helsinki and informed consent was obtained from all study participants, ethical approval was obtained from the SingHealth centralized Singapore Eye Research Institute Institutional Review Board.

### Ophthalmic examinations

Each participant underwent a standardized eye examination, of which the relevant tests are described herein. Visual acuity, subjective refraction, and axial length measurements (IOL Master V3.01; Carl Zeis Meditec AG, Jena, Germany) were measured by research optometrists and trained research coordinators. Slit-lamp biomicroscopy (model BQ-900; Haag-Streit, Switzerland), intraocular pressure (IOP) by Goldmann applanation tonometer (GAT; Haag-Streit, Bern, Switzerland), anterior segment photography (slit lamp assisted biomicroscopy of the anterior segment, Topcon model DC-1 with FD-21 flash attachment; Topcon, Tokyo, Japan), and post dilation fundoscopy was performed by study ophthalmologists. To ensure adequate anterior segment photographs, the slit beam width was 2 mm, height was adjusted to reach the edges of the dilated pupil on both ends of the beam vertically bisecting the lens at a 45-degree angle, the flash power 6 and magnification 16 × . The photograph was focused on cornea.

### Pterygium definition and grading

A pterygium is defined as a fibrovascular subepithelial growth extending across the limbus onto the clear cornea. A known simple pterygium grading system^13,14^ based on morphologic features found on slit lamp examination was used. In brief, there were 3 grades of pterygia. Grade 1 (transparent) was the least severe and denotes a pterygium in which the episcleral vessels underlying the body of the pterygium were unobscured and can be clearly distinguished. Grade 3 (opaque) was the most severe and denotes a thick, fleshy pterygium in which episcleral vessels underlying the body of the pterygium are completely obscured. Pterygia that presented in between the descriptions of grade 1 and 3 were labelled as grade 2 (intermediate).

For each eye, the pterygium was graded by the study ophthalmologist (XL Fang) based on anterior segment photographs taken at baseline and at the 6-year follow-up visit. Any uncertainty was discussed between two study ophthalmologists (XL Fang, and S Thakur) and adjudicated by the senior author (C-Y Cheng). Before the grading, a pilot study was conducted to assess the intra-grader and inter-grader variability of pterygium grading. Two study ophthalmologists (XL Fang, and S Thakur) performed the grading on 50 anterior segment photos. After two weeks, these photos were graded again by one of the study ophthalmologists (XL Fang). The results showed that our grading had a good intra-grader agreement of 0.87 (95% CI 0.83–0.97), and a substantial inter-grader agreement of 0.80 (95% CI 0.65–0.95).

Incident pterygium was defined as patients with no visible pterygium on anterior segment photograph or history of pterygium removal surgery at baseline visit, a new pterygium or history of pterygium removal surgery that was presented at the 6-year follow-up visit in either eye. Pterygium progression was defined as patients who progressed from unilateral to bilateral pterygium 6 years later or eyes progressed from grade 1 or 2 pterygium to grade 3 pterygium or surgically removed pterygium 6 years later.

### Other measurements and definitions

Interviewer-administered questionnaires were conducted in participants’ language of choice (English, Chinese, Malay, or Tamil). Information collected from interviewer administered questionnaires included socioeconomic status (such as education level and income levels), occupation (predominantly indoor or outdoor), medical history, medication use, alcohol consumption, and smoking status^15^. Body mass index (BMI) was calculated as body weight (in kilograms) divided by body height (in metres) squared and categorized as either underweight (< 18.5 kg/m^2^), normal (≥ 18.5 kg/m^2^ but < 25 kg/m^2^), overweight (≥ 25 kg/m^2^ but < 30 kg/m^2^) or obese (≥ 30 kg/m^2^)^12^. Blood pressure (BP) was measured using a digital automatic BP monitor (Dinamap model Pro100V2; Criticon GmbH, Norderstedt, Germany)^16^. Hypertension was defined as either systolic blood pressure ≥ 140 mm Hg, diastolic blood pressure ≥ 90 mm Hg, current use of blood pressure medication or self-reported history of hypertension^16^. Smoking status was defined as current and previous history of smoking. Educational level was categorized as no formal education (≤ 6 years, primary or lower), formal education (≥ 7 years, including university education)^12^. Individual monthly income was based on Singapore dollars (SGD) and was categorized as lower monthly income(< S$2000), middle or higher monthly income(≥ S$2000)^12^. Non-fasting venous blood samples were collected for measurement of plasma cholesterol [total cholesterol, low-density lipoprotein (LDL) and high-density lipoprotein (HDL)], serum triglyceride (TG), HbA1c, and random serum glucose. Diabetes was defined as either random glucose ≥ 11.1 mmol/l, HbA1c ≥ 6.5%, current use of diabetic medication or self-reported history of diabetes^16^. Hyperlipidaemia was defined as either total cholesterol ≥ 6.2 mmol/L, current use of lipid-lowering medication or self-reported history of hyperlipidaemia^16^.

### Statistical analysis

Patients with anterior segment photographs taken in both eyes at both baseline and follow-up studies were included. Participants with pterygium in either eye at baseline were excluded.

All statistical analyses were performed using R language (R V. 3.5.3, R Foundation for statistical computing 2019, Vienna, Austria). Age and BMI were analysed as continuous variables while gender, ethnicity, occupation, education level, monthly income, current smoking status, alcohol consumption, presence of hypertension, diabetes, and hyperlipidemia were analysed as categorical variables. For comparison of subjects’ characteristics, independent t-tests were performed for continuous variables, and chi-squared tests was performed for categorical variables. Age-adjusted pterygium incidence for the entire SEED sample and individual ethnic groups were calculated and standardization to the 2010 Singapore Population Census. Cumulative incidence was calculated based on the development of pterygium in either eye. Multivariable logistic regression model was established to calculate the odds ratios (ORs) and 95% confidence intervals (CIs), using incident pterygium as the outcome measure and various potential predictors as exposures. In all statistical analyses, a *P* value of less than 0.05 was considered statistically significant.

## Results

**Figure 1:**
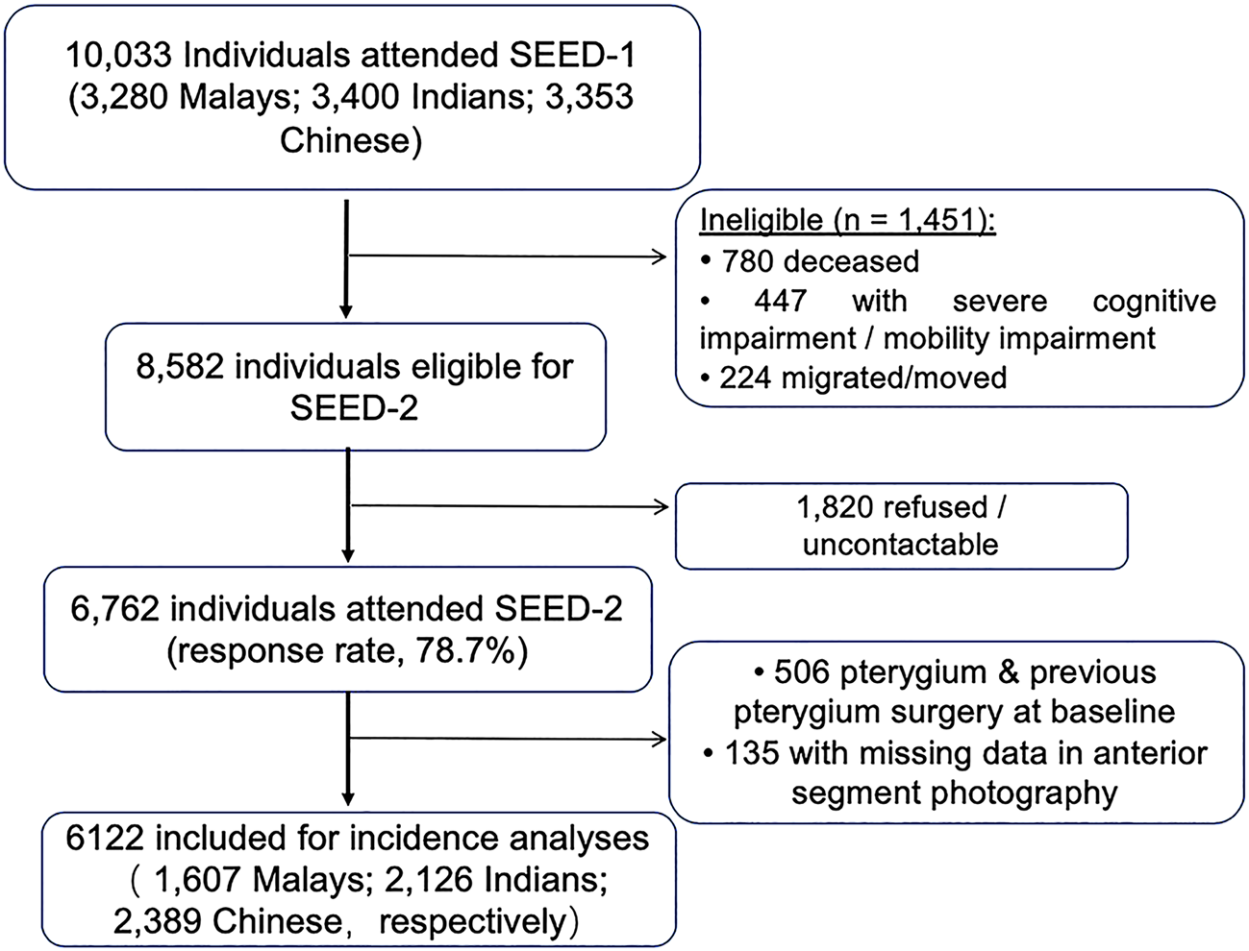
Flow chart that illustrates the subject selection process for this study.

A total of 6,762 (78.7% of eligible) participants from the baseline SEED study were re-examined at the 6-year follow-up visit (Fig. 1). Of the 6,762 subjects (1,901 Malays, 2,200 Indians and 2,661 Chinese) that were re-examined, we excluded 135 participants who did not have anterior segment photographs taken in both eyes, and 506 participants who were diagnosed with pterygium or previous pterygium surgery in either eye at baseline visit. We included a final sample of 6,122 participants (1,607 Malays, 2,126 Indians and 2,389 Chinese) without pterygium at baseline who were at risk of incident pterygium in either eye (Fig. 1).

When comparing baseline characteristics between participants and non-participants at the 6-year follow-up examinations, non-participants were older, more likely to be men, more likely to be current smokers, more likely to have no formal education, outdoor occupation, lower monthly income, alcohol consumption, hypertension, and diabetes(all *P* ≤ 0.007), Supplemental Table 3).

The overall 6-year incidence of pterygium was 1.4% (2.1% in Chinese, 1.6% in Malay, 0.4% in Indian). In 73 (88.0%) of the 83 subjects, the incident pterygium had occurred unilaterally, whereas in 10 (12.0%) subjects, the incident pterygium had developed bilaterally. We compared baseline characteristics between participants with and without incident pterygium and those without pterygium at the 6-year follow-up examination. Participants with incident pterygium were older, more likely to be men, more likely to have outdoor occupations, lower monthly income, and more likely to have hypertension (Table 1). Overall, males had slightly higher incidence rate of pterygium compared to females (Supplemental Table 4).

**Table 1:** Comparison of baseline characteristics between participants with and without incident pterygium.

	Combined Singapore Epidemiology of Eye Diseases Study (n = 6122)		
Baseline factors	Participants with incident pterygium	Participants without pterygium	*P* value
Total no	83	6,039	
Age (yrs), mean (SD)	60.7 (7.85)	56.73 (9.27)	** < 0.001**
Male, n (%)	50 (60.2)	2,802 (46.4)	**0.016**
Body mass index (BMI, kg/m^2^)	24.8 (4.19)	25.4 (4.48)	0.170
Current smokers, n (%)	7 (8.4)	861 (14.3)	0.175
Formal education, n (%)	62 (74.7)	4,954 (82.1)	0.110
Outdoor occupation, n (%)	13 (15.7)	406 (6.7)	**0.003**
Lower monthly income < S$2000,n (%)	67 (82.7)	4,255 (72.1)	**0.046**
Alcohol consumption, n (%)	7 (8.4)	559 (9.3)	0.945
Hypertension, n (%)	59 (72)	3,497 (58)	**0.015**
Diabetes, n (%)	17 (20.5)	1,578 (26.1)	0.299
Hyperlipidemia, n (%)	41 (50)	2,597 (44.1)	0.339

*P* value was calculated based on chi-square test or independent t-test, where appropriate.

The overall age-adjusted 6-year incidence was 1.2% (95% CI 1.0%-1.6%; Table 2). The age-adjusted 6-year incidence for Chinese was 1.9% (95% CI 1.4%-2.5%), Malay was 1.4% (95% CI 0.9%-2.1%), and Indian was 0.3% (95% CI 0.3%-0.7%) (Table 2). The incidence in Indians was the lowest and was significantly different from that in Malays (*P* = 0.005) and Chinese (*P* < 0.001; Table 2). We observed an increasing trend in incidence with age, from 0.3% among those 40 to 49 years, and up to 2.1% among those 70 years or older(*P* < 0.001).

**Table 2:** Incident pterygium stratified by age groups and across the ethnic groups of Chinese, Malays, and Indians.

Baseline age group	Chinese		Malays		Indians		Overall	
	No./Total*	Incidence (%)	No./Total*	Incidence (%)	No./Total*	Incidence (%)	No./Total*	Incidence (%)
40–49	4/587	0.7	1/504	0.2	0/660	0.0	5/1,751	0.3
50–59	22/910	2.4	14/572	2.4	1/731	0.1	37/2,213	1.7
60–69	16/608	2.6	6/339	1.8	5/541	0.9	27/1,488	1.8
≥ 70	7/284	2.5	4/192	2.1	3/194	1.5	14/670	2.1
*P* value (for trend)^§^		0.036		0.051		< 0.001		< 0.001
Crude	49/2,389	2.1	25/1,607	1.6	9/2,126	0.4	83/6,122	1.4
Age-adjusted (95%CI)^†^		1.9 (1.4–2.5)^‡^		1.4 (0.9–2.1)^‡^		0.3 (0.1–0.7)^‡^		1.2 (1.0–1.6)

*CI* confidence interval.

*Number of cases/numbers at risk.

^§^Cochran—Armitage trend test.

^†^Standardized to 2010 Singapore population census.

^‡^*P* values for age-adjusted incidence between the 3 ethnic groups are: Chinese vs. Indians, *P* < 0.001; Malays vs. Indians, *P* = 0.005; Chinese vs. Malays, *P* = 0.771.

Table 3 shows the 6-year progression of pterygium. At baseline, there were 329 participants [5.3%] with unilateral pterygium [165 Malay participants (8.4%), 43 Indian participants (2.0%), and 121 Chinese (4.5%)]. Of the 329 participants, 14 (4.3%(95%CI, 2.1–6.5); 8 Chinese, 5 Malays, and 1 Indian) further developed pterygium in the fellow eye over 6 years.). On the other hand, amongst the 604 eyes which presented with grade 1 or 2 pterygium at baseline, 41(6.8%(95%CI, 4.8–8.8); 21 Chinese, 15 Malays and 5 Indian eyes) progressed to grade 3 or underwent pterygium removal surgery during the 6-year follow up visit.

**Table 3:** Progression of Pterygium across the ethnic groups of Chinese, Malays, and Indians.

	Six-year progression		
	(A) Person-specific (unilateral pterygium at baseline to bilateral pterygium six years later)		
	No. unilateral pterygium at baseline	No. bilateral pterygium six years later	%^†^ (95% Confidence Interval)
Chinese	121	8	6.6 (2.2–11.0)
Malay	165	5	3.0 (0.4–5.6)
Indian	43	1	2.3 (0.0–6.8)
Overall	329	14	4.3 (2.1–6.5)

	Six-year progression		
	(B) Eye-specific (mild pterygium [grade 1 & 2] at baseline to severe pterygium [grade 3 or surgically removed] six years later)		
	No. mild pterygium	No. severe pterygium	%^‡^ (95% Confidence Interval)
Chinese	206	21	10.2 (6.1–14.3)
Malay	327	15	4.6 (2.3–6.9)
Indian	71	5	7.0 (1.1–12.9)
Overall	604	41	6.8 (4.8–8.8)

(A)†Percentage of subjects who progressed from unilateral at baseline to bilateral pterygium six-year later.

^‡^*P* values for progression rate comparisons between ethnic groups: Chinese vs. Malay, *P* = 0.164; Chinese vs. Indians, *P* = 0.448; Malay Vs Indian, *P* = 1.000.

(B)‡ percentage of eyes with progressed from grade 1 or 2 pterygium at baseline to grade 3 or surgically removed pterygium six-year later.

^‡^*P* values for progression rate comparisons between ethnic groups: Chinese vs. Malay, *P* = 0.019; Chinese vs. Indian, *P* = 0.637; Malay Vs Indian, *P* = 0.374.

In multivariable regression analysis, after adjusting for baseline age, gender, ethnicity, BMI, occupation, education level, monthly income, current smoking status, alcohol consumption, hypertension, diabetes and hyperlipidemia, the Chinese (odds ratio [OR] = 4.21; 95% confidence interval [CI] 2.12–9.35; *P* < 0.001) and Malays (OR 3.22; 95% CI 1.52–7.45; *P* = 0.004) were more likely to develop pterygium, as compared to Indians (Table 4). There was no significant difference between Chinese and Malays (*P* = 0.336). In addition, the male gender (OR 2.13; 95% CI 1.26–3.63; *P* = 0.005) and an outdoor occupation (OR 2.33; 95% CI 1.16–4.38; *P* = 0.012) were associated with a higher risk of developing pterygium. Current smoking status at baseline was associated with a lower risk of developing pterygium (OR 0.41; 95% CI 0.16–0.87, *P* = 0.032). Age was not significantly associated with incident pterygium (*P* = 0.083) in this multivariable regression analysis.

**Table 4:** Multivariable logistic regression analysis on the association between baseline factors and incident pterygium.

Baseline Factors	Odds ratio (95% Confidence Interval)	*P* value
Age per decade	1.03 (1.00–1.06)	0.083
Male (vs female)	2.13 (1.26–3.63)	**0.005**
**Ethnicity**		
Indian	Reference	
Chinese	4.21 (2.12–9.35)	** < 0.001**
Malay	3.22 (1.52–7.45)	**0.004**
**BMI categories**		
Normal (18.5 ≤ BMI < 25)	Reference	
Underweight (BMI < 18.5)	1.92 (0.65–4.64)	0.184
Overweight (25 ≤ BMI < 30)	1.17 (0.70–1.94)	0.538
Obese (BMI ≥ 30)	1.02 (0.43–2.20)	0.953
Outdoor occupation(Indoor as reference)	2.33 (1.16–4.38)	**0.012**
No formal education	1.21 (0.67–2.14)	0.515
Lower monthly income < S$2000	1.74 (0.94–3.41)	0.091
Current smoking status, yes	0.41 (0.16–0.87)	**0.032**
Alcohol consumption, yes	1.03 (0.41–2.21)	0.945
Hypertension, yes	1.33 (0.78–2.33)	0.307
Diabetes, yes	0.70 (0.39–1.22)	0.229
Hyperlipidemia, yes	1.16 (0.73–1.86)	0.523

*BMI* body mass index.

## Discussion

In this population-based cohort study of three major ethnic groups in Asia, the overall age-adjusted 6-year incidence for pterygium was 1.2%, with Chinese having a slightly higher incidence (1.9%) compared to Malays (1.4%) and Indians (0.3%). We observed that Chinese and Malays (compared to Indians), male gender, outdoor occupation, and no current smoking status were associated with higher risk of developing pterygium. To our best knowledge, this large multi-ethnic Asian population-based study conducted in a standardized manner, provides the first population data for direct comparison of ethnic differences for incidence of pterygium. Our findings will be useful in further identifying elderly who may be at higher risk of developing pterygium for patient education in a bid to further prevent onset of pterygium.

In this study, we found that Chinese and Malays were more likely to develop pterygium compared to Indians. Our findings are consistent with other studies which reported that darker skin pigmentation was protective against pterygium^3,17,18^. Persons with darker skin pigmentation are known to have more melanin in their skins (and correspondingly with higher conjunctival pigmentation level), and thus may be more protective against UV exposure^19^. Notably, we observed that Chinese have the highest incidence of pterygium (1.9%). In a standardized setting and one that was conducted in the equatorial region, we observed that Chinese had higher incidence of pterygium compared to Indians and Malays. Coupling with this finding, it is thus plausible that Chinese residing in areas with higher UV exposures (e.g.Tibet, Yunnan) may have even higher risk of developing pterygium. This was further supported by previous studies which found high prevalence of pterygium in Yunnan study^20,21^. Taken together, the higher incidence observed in Chinese populations is potentially due to combined elements of genetic and environmental factors (i.e .UV-exposures).

On the other hand, it is however interesting to note that in the Blue Mountain Eye study, dark skin colour was associated with higher odds of having pterygium^22^. Nevertheless, it should be noted that the finding was based on a cross-sectional evaluation, using univariate analysis which did not take into account other important risk factors such as age and outdoor occupation, and thus should be evaluated with care. Furthermore, there were small number of participants with dark skin (mainly Aborigines) in that study. The combined factors that Australia has high UV levels and Aborigines tend to lead a more outdoor lifestyle, might also explain this observation^22^.

We observed an increasing trend of incident pterygium with age. The incidence rate increased from 0.3% among those 40 to 49 years to 2.1% among those 70 years or older (*P* < 0.001). Most previous cross-sectional studies had reported significant association between older age and presence of pterygium^23–25^.

Another factor thought to be associated with pterygium is male gender, a twofold increased risk was noted for men in this incidence investigation and a fivefold increased risk reported for men in the SEED baseline study^26^. This is in line with most prevalence studies^23,24,27,28^ which suggested male gender as an important risk factor. As outdoor professions are typically done by men, and outdoor occupations and UV exposures are also known risk factors for pterygium, this may explain the observed higher risk in men. On the other hand, there were two other cohort studies (Yunnan and South Korea) which observed female gender to be associated with higher risk of developing pterygium^5,6^.

In this current study, while all subjects lived in the same socioeconomic urban setting, we found that an outdoor occupation was still associated with a higher risk of developing pterygium. Study subjects with occupations which were primarily outdoors were 2 times more likely to developing pterygium in 6 years compared to those who worked indoors after adjusting for baseline age, gender, and ethnicity. This is in agreement with the Barbados Eye Study^3^ and Yunnan Minority Eye Study^5^ which also reported outdoor occupation as a predictive factor for pterygium. Likewise, The Russian Ural Eye and Medical Study^25^, the Beijing Eye Study^4^ and Korean cohort study^6^ observed that subjects living in a relatively rural area were associated with increased risks of pterygium.

Current smoking status was protective for pterygium in this study. This is in line with the Korea longitudinal cohort study^29^, cigarette smoking was associated with a reduced risk of pterygium, and this protective effect was more pronounced among current smokers than among past smokers. However, in another two longitudinal cohort studies, the BES study^3^ and Yunnan Minority Eye Study^5^ did not observe significant associations between past smokers and incident pterygium, but reported a relatively weaker protective effect (OR 0.82, OR 0.66, respectively) between smoking and incident of pterygium, albeit being non-significant. Previous cross-sectional population-based studies have reported inconsistent results regarding the prevalence of pterygium in individuals who smoke. There were other studies which reported that smokers were less likely to have pterygium^17,30–35^. On the contrary, Li et al. reported smoking as a risk factor for pterygium among Chinese^36^. Although the protective effect of current smoking status is significant (*P* = 0.032) in our study, it is pertinent to note that the analysis only included 7 positive incident cases with positive smoking status at baseline. Given the few numbers, the effects of smoking on pterygium cannot be concluded definitively. Hence, future studies are necessary to ascertain this aspect.

Our study provided a unique opportunity to examine possible ethnic differences in pterygium incidence in Asians, in a relatively common geographic and socioeconomic environment. The other strengths of this study include masked grading of anterior segment photographs from both eyes, based on a standardised protocol, large sample size, and a comprehensive measurement of systemic and ocular parameters allowing multiple relevant confounders to be accounted for in the analysis.

However, this study also has a few limitations. First, there was a limited number of incident pterygium cases, thus finer stratified analysis or interaction analysis could not be performed due to limited power. Second excluded individuals had slightly different baseline characteristics as compared with included subjects (Supplemental Table 3). In general, excluded subjects were older, more likely to have been current smokers, more likely to have no formal education, outdoor occupation, lower monthly income, alcohol consumption, and more likely to have hypertension, diabetes(all *P* ≤ 0.007), thus potential follow-up bias cannot be entirely ruled out in our sample. Third, the follow-up time differed slightly among the three ethnic groups with the mean follow-up time being 6.63 years in Malays, 6.05 years in Indians, 5.83 years in Chinese. Nevertheless, even further adjusting for follow up time in our multivariable model, it was still observed that Chinese have higher incidence of pterygium compared to Indians (OR 4.45; *P* < 0.001, data not shown in tables), and Malays (OR 1.66; *P* = 0.083, data not shown in tables).

In conclusion, we observed that Chinese and Malays were more likely to develop pterygium compared to Indians over 6 years. In addition, male gender, outdoor occupation, and non-smokers were associated with higher risk of developing pterygium after accounting for other common risk factors. These results provide some new findings for pterygium in Asian populations, which will be useful in further identifying who may be at higher risk of developing pterygium.

## Supplementary information

Supplementary Information 1.

## References

[1] Coster D (1995). Pterygium—an ophthalmic enigma. Br. J. Ophthalmol..

[2] Tomidokoro A (2000). Effects of pterygium on corneal spherical power and astigmatism. Ophthalmology.

[3] Nemesure B, Wu SY, Hennis A, Leske MC, Barbados Eye Studies, G (2008). Nine-year incidence and risk factors for pterygium in the barbados eye studies. Ophthalmology.

[4] Zhao L (2013). 10-year incidence and associations of pterygium in adult Chinese: the Beijing Eye Study. Invest. Ophthalmol. Vis. Sci..

[5] Li L (2015). Five-year incidence and predictors for Pterygium in a Rural Community in China: the Yunnan minority eye study. Cornea.

[6] Rim TH, Kang MJ, Choi M, Seo KY, Kim SS (2017). The incidence and prevalence of pterygium in South Korea: a 10-year population-based Korean cohort study. PLoS ONE.

[7] Fenwick EK (2016). Association of changes in visual acuity with vision-specific functioning in the Singapore Malay Eye Study. JAMA Ophthalmol..

[8] Lavanya R (2009). Methodology of the Singapore Indian Chinese Cohort (SICC) Eye Study: quantifying ethnic variations in the epidemiology of eye diseases in Asians. Ophthalmic Epidemiol..

[9] Gupta P (2013). Determinants of macular thickness using spectral domain optical coherence tomography in healthy eyes: the Singapore Chinese Eye study. Invest. Ophthalmol. Vis. Sci..

[10] Rosman M (2012). Singapore Malay Eye Study: rationale and methodology of 6-year follow-up study (SiMES-2). Clin. Exp. Ophthalmol..

[11] Sabanayagam C (2017). Singapore Indian Eye Study-2: methodology and impact of migration on systemic and eye outcomes. Clin. Exp. Ophthalmol..

[12] Majithia, S.*, et al.* Singapore Chinese Eye Study: key findings from baseline examination and the rationale, methodology of the 6-year follow-up series. *Br J Ophthalmol* (2019). 10.1136/bjophthalmol-2019-314760 31401553

[13] Saw SM, Tan D (1999). Pterygium: prevalence, demography and risk factors. Ophthalm. Epidemiol..

[14] Tan DT, Chee SP, Dear KB, Lim AS (1997). Effect of pterygium morphology on pterygium recurrence in a controlled trial comparing conjunctivalautografting with bare sclera excision. Arch. Ophthalmol..

[15] Tan AG (2018). Six-year incidence of and risk factors for cataract surgery in a multi-ethnic Asian population: the Singapore Epidemiology of Eye Diseases Study. Ophthalmology.

[16] Tan GS (2018). Ethnic differences in the prevalence and risk factors of diabetic retinopathy: the Singapore Epidemiology of Eye Diseases Study. Ophthalmology.

[17] Luthra R (2001). Frequency and risk factors for pterygium in the Barbados Eye Study. Arch. Ophthalmol..

[18] Mackenzie FD, Hirst LW, Battistutta D, Green A (1992). Risk analysis in the development of pterygia. Ophthalmology.

[19] Stacey A, Rajaii F, Schmidt-Erfurth U, Kohnen T (2016). Pigmented lesions of the conjunctiva. Encyclopedia of Ophthalmology.

[20] Zhong H (2012). Prevalence of and risk factors for pterygium in rural adult chinese populations of the Bai nationality in Dali: the Yunnan Minority Eye Study. Invest. Ophthalmol. Vis. Sci..

[21] Zhong H (2016). Ethnic variations in pterygium in a rural population in Southwestern China: the Yunnan Minority Eye Studies. Ophthalm. Epidemiol..

[22] Panchapakesan J, Hourihan F, Mitchell P (1998). Prevalence of pterygium and pinguecula: the Blue Mountains Eye Study. Aust. N. Z. J. Ophthalmol..

[23] Xu Y (2013). Automatic Grading of Nuclear Cataracts from Slit-Lamp Lens Images Using Group Sparsity Regression.

[24] Rezvan F (2018). Prevalence and risk factors of pterygium: a systematic review and meta-analysis. Surv. Ophthalmol..

[25] Bikbov MM (2019). Pterygium prevalence and its associations in a Russian Population: the ural eye and medical study. Am. J. Ophthalmol..

[26] Ang M (2012). Prevalence of and racial differences in pterygium: a multiethnic population study in Asians. Ophthalmology.

[27] Shiroma H (2009). Prevalence and risk factors of pterygium in a southwestern island of Japan: the Kumejima Study. Am. J. Ophthalmol..

[28] Anbesse DH (2017). Prevalence and associated factors of pterygium among adults living in Gondar city, Northwest Ethiopia. PLoS ONE.

[29] Rim TH, Kim DW, Cheng C-Y, Kim SS (2017). Protective effect of smoking against pterygium development in men: a nationwide longitudinal cohort study in South Korea. BMJ Open.

[30] Gazzard G (2002). Pterygium in Indonesia: prevalence, severity and risk factors. Br. J. Ophthalmol..

[31] West S, Munoz B (2009). Prevalence of pterygium in Latinos: Proyecto VER. Br. J. Ophthalmol..

[32] Sun LP (2013). The prevalence of and risk factors associated with pterygium in a rural adult Chinese population: the Handan Eye Study. Ophthalm. Epidemiol..

[33] Pan Z (2019). Prevalence and risk factors for pterygium: a cross-sectional study in Han and Manchu ethnic populations in Hebei, China. BMJ Open.

[34] McCarty CA, Fu CL, Taylor HR (2000). Epidemiology of pterygium in Victoria Australia. Br. J. Ophthalmol..

[35] Rim TH, Nam J, Kim EK, Kim TI (2013). Risk factors associated with pterygium and its subtypes in Korea: the Korean National Health and Nutrition Examination Survey 2008–2010. Cornea.

[36] Li Z, Cui H (2013). Prevalence and associated factors for pterygium in a rural adult population (the Southern Harbin Eye Study). Cornea.

