# Supplementary material for: Ethnic differences in the incidence of pterygium in a multi-ethnic Asian population: the Singapore Epidemiology of Eye Diseases Study
Source: Sci Rep. 2021 Jan 12;11:501. doi: 10.1038/s41598-020-79920-9 (PMC7804407; doi:10.1038/s41598-020-79920-9)
Supplement: Supplementary file 1 — Supplementary Information 1. [file 41598_2020_79920_MOESM1_ESM.docx]

**Ethnic Differences in the Incidence of pterygium in a Multi-Ethnic Asian Population: The Singapore Epidemiology of Eye Diseases Study**

Xiaoling Fang^1,2^, Crystal Chong Chun Yuen ^1^, Sahil Thakur^1^，Zhi Da Soh^1^, Zhen Ling Teo^1^, Shivani Majithia^1^, Zhi Wei Lim^1^, Tyler Hyungtaek^1^, Charumathi Sabanayagam^1,3,4^, Tien Yin Wong^1,3,4^, Ching-Yu Cheng ^1,3,4#^, Yih-Chung Tham^1,3#^

^1^Ocular Epidemiology, Singapore Eye Research Institute, Singapore National Eye Centre, Singapore

^2^Department of Ophthalmology，Shanghai Eye Diseases Prevention &Treatment Center/ Shanghai Eye Hospital，Shanghai, China

^3^Ophthalmology & Visual Sciences Academic Clinical Program (Eye ACP), Duke-NUS Medical School, Singapore

^4^Department of Ophthalmology, Yong Loo Lin School of Medicine, National University of Singapore, Singapore

^#^Contributed equally

**Supplemental table 1.** Summary on Prevalence with Pterygium in Population-Based Cross-Sectional Studies Around the World.

| No. | First Author | Publication year | Country | Ethnic | Sample size | Age (years) | Crude Prevalence (%) |
| --- | --- | --- | --- | --- | --- | --- | --- |
| 1 | Landers^1^ | 2011 | Australia | Australian | 1,884 | ≥20 | 7.8 |
| 2 | Luthra^2^ | 2001 | Barbados | Barbadian | 2,617 | 40-84 | 23.4 |
| 3 | Lu^3^ | 2007 | China (Tibtan) | Chinese | 2,229 | ≥40 | 14.49 |
| 4 | Lu^4^ | 2009 | China (Henan) | Chinese | 2,112 | ≥40 | 17.9 |
| 5 | Ma^5^ | 2007 | China (Beijing) | Chinese | 4,439 | ≥40 | 2.88 |
| 6 | Pan^6^ | 2019 | China (Hebei) | Chinese | 3,790 | ≥40 | 6.1 |
| 7 | Chen^7^ | 2015 | China (Xinjiang) | Chinese | 4,617 | 30-80 | 11.8 |
| 8 | Jiao^8^ | 2014 | China (Shandong) | Chinese | 17,816 | ≥50 | 10.53 |
| 9 | Li^9^ | 2013 | China (Harbin) | Chinese | 5,057 | 50-96 | 6.38 |
| 10 | Li^10^ | 2014 | China (Heilongjian) | Chinese | 10,384 | 18-94 | 2.5 |
| 11 | Sun^11^ | 2013 | China (Handan) | Chinese | 6,685 | ≥40 | 5.99 |
| 12 | Zhong^12^ | 2012 | China (Yunnan) | Chinese | 2,133 | ≥50 | 39 |
| 13 | Zhong^13^ | 2016 | China (Yunnan) | Chinese | 6,418 | ≥40 | 39.5 |
| 14 | Asokan^14^ | 2012 | India | Indian | 7,774 | ≥40 | 9.5 |
| 15 | Nangia^15^ | 2013 | India | Indian | 4,711 | 30-85 | 12.9 |
| 16 | Marmamula^16^ | 2013 | India | Indian | 5,586 | ≥30 | 11.7 |
| 17 | Gazzard^17^ | 2002 | Indonesia | Malay/Indonesians | 1,210 | ≥21 | 9.3 |
| 18 | Fotouhi^18^ | 2009 | Iran (Tehran) | Persian | 4,564 | All ages | 1.3 |
| 19 | Rezvan^19^ | 2012 | Iran (Shahroud) | Iranian | 5,190 | 40-64 | 9.4 |
| 20 | Nemet^20^ | 2014 | Israel | Israelis | 16,054 | 18-22 | 25.14 |
| 21 | Tano^21^ | 2013 | Japan | Japanese | 2,312 | 40-74 | 4.4 |
| 22 | Lim^22^ | 2015 | Korea | Korean | 13,024 | ≥40 | 6.22 |
| 23 | Pyo^23^ | 2016 | Korea | Korean | 9,193 | ≥40 | 8.8 |
| 24 | Rim^24^ | 2013 | Korea | Korean | 14,920 | ≥30 | 6.7 |
| 25 | Durkin^25^ | 2007 | Myanmar | Burmese | 2,076 | ≥40 | 19.6 |
| 26 | Mukharran^26^ | 2019 | Russia | Russians, Bashkirs, Tatars, Ukrainians, | 5,899 | ≥40 | 2.3 |
| 27 | Ang^27^ | 2012 | Singapore | Indian,Malay,Chinese | 8,874 | 40-80 | 10.14 |
| 28 | Cajucom-Uy^28^ | 2010 | Singapore | Malay | 3,280 | 40-80 | 15.6 |
| 29 | Wong^29^ | 2001 | Singapore | Chinese | 1,232 | 40-79 | 9.7 |
| 30 | West^30^ | 2009 | USA (Arizona) | Hispanics | 4,767 | ≥40 | 16.2 |
| 31 | McCarty^31^ | 2000 | Victoria | Australian | 5,147 | ≥40 | 2.83 |

S**upplemental table 2.** Incidence of pterygium among different population-based studies of similar age ranges.

| Study | Follow-up (years) | Age (years) | Sample size | Incidence of pterygium |
| --- | --- | --- | --- | --- |
| Barbados Eye Study | 9 | 40-84 | 1,888 | 11.6% |
| Beijing Eye Study | 10 | ≥40 | 2,695 | 4.9% |
| Korean cohort study | 10 | ≥40 | 4,595,643 | 4.3% |
| Yunnan Minority Eye Study | 5 | ≥50 | 1,520 | 6.8% |

**Supplemental table 3**. Baseline Characteristics of Participants and Nonparticipants in 6-Year Follow-up Examinations from the Singapore Epidemiology of Eye Diseases Study Ethnic Groups.

|  | Combined Singapore Epidemiology of Eye Diseases Study (n=10033) | | |
| --- | --- | --- | --- |
| Baseline Factors | Participants | Nonparticipants | P value |
| Total no. | 6762 | 3,271 |  |
| Age (yrs), mean (SD) | 57.37 (9.47) | 61.97 (11.43) | **<0.001** |
| Male, n (%) | 3,247 (48.0) | 1,696 (51.8) | **<0.001** |
| Body mass index (BMI, kg/m^2^) | 25.34 (4.46) | 25.52 (5.17) | 0.080 |
| Current smokers, n (%) | 972 (14.4) | 631 (19.3) | **<0.001** |
| Formal education, n (%) | 5,447 (80.6) | 2,233 (68.4) | **<0.001** |
| Outdoor occupation, n (%) | 470 (7.0) | 175 (5.4) | **0.003** |
| Lower monthly income＜S$2000,n (%) | 4,853 (73.4) | 2,773 (86.5) | **<0.001** |
| Alcohol consumption, n (%) | 608 (9.0) | 240 (7.4) | **0.007** |
| Hypertension, n (%) | 4,024 (59.7) | 2,369 (72.6) | **<0.001** |
| Diabetes, n (%) | 1,770 (26.2) | 1,194 (36.5) | **<0.001** |
| Hyperlipidemia, n (%) | 2,964 (45.0) | 1,445 (46.4) | 0.182 |

P value was calculated based on chi-square test or independent t-test, where appropriate.

**Supplemental table 4**. Incident pterygium stratified by gender and across the ethnic groups of Chinese, Malays, and Indians.

|  | **Chinese** | | **Malay** | | **Indian** | | **Overall** | |
| --- | --- | --- | --- | --- | --- | --- | --- | --- |
|  | No./ Total^*^ | Incidence (%) | No./ Total^*^ | Incidence (%) | No./ Total^*^ | Incidence (%) | No./ Total^*^ | Incidence (%) |
| Female | 17/ 1,274 | 1.3 | 12/ 913 | 1.3 | 4/ 1,083 | 0.4 | 33/ 3,270 | 1.0 |
| Male | 32/ 1,115 | 2.9 | 13/ 694 | 1.9 | 5/ 1,043 | 0.5 | 50/ 2,852 | 1.8 |

Reference for Supplemental table 1:

1 Landers, J., Henderson, T. & Craig, J. Prevalence of pterygium in indigenous Australians within central Australia: the Central Australian Ocular Health Study. *Clinical & Experimental Ophthalmology* **39**, 604-606, doi:10.1111/j.1442-9071.2011.02532.x (2011).

2 Luthra, R. *et al.* Frequency and risk factors for pterygium in the Barbados Eye Study. *Arch Ophthalmol* **119**, 1827-1832, doi:10.1001/archopht.119.12.1827 (2001).

3 Lu, P. *et al.* Pterygium in Tibetans: a population-based study in China. *Clin Exp Ophthalmol* **35**, 828-833, doi:10.1111/j.1442-9071.2007.01630.x (2007).

4 Lu, J. *et al.* Pterygium in an aged Mongolian population: a population-based study in China. *Eye (Lond)* **23**, 421-427, doi:10.1038/sj.eye.6703005 (2009).

5 Ma, K., Xu, L., Jie, Y. & Jonas, J. B. Prevalence of and factors associated with pterygium in adult Chinese: the Beijing Eye Study. *Cornea* **26**, 1184-1186, doi:10.1097/ICO.0b013e318151f9c6 (2007).

6 Pan, Z. *et al.* Prevalence and risk factors for pterygium: a cross-sectional study in Han and Manchu ethnic populations in Hebei, China. *BMJ Open* **9**, e025725, doi:10.1136/bmjopen-2018-025725 (2019).

7 Chen, T. *et al.* Prevalence and racial differences in pterygium: a cross-sectional study in Han and Uygur adults in Xinjiang, China. *Invest Ophthalmol Vis Sci* **56**, 1109-1117, doi:10.1167/iovs.14-15994 (2015).

8 Jiao, W. *et al.* Prevalence and risk factors for pterygium in rural older adults in Shandong Province of China: a cross-sectional study. *Biomed Res Int* **2014**, 658648, doi:10.1155/2014/658648 (2014).

9 Li, Z. & Cui, H. Prevalence and associated factors for pterygium in a rural adult population (the Southern Harbin Eye Study). *Cornea* **32**, 806-809, doi:10.1097/ICO.0b013e31826dff30 (2013).

10 Li, Z. *et al.* Prevalence of and risk factors for pterygia in a rural Northern Chinese population. *Ophthalmic Epidemiol* **21**, 378-383, doi:10.3109/09286586.2014.967359 (2014).

11 Sun, L. P. *et al.* The prevalence of and risk factors associated with pterygium in a rural adult Chinese population: the Handan Eye Study. *Ophthalmic Epidemiol* **20**, 148-154, doi:10.3109/09286586.2013.763991 (2013).

12 Zhong, H. *et al.* Prevalence of and risk factors for pterygium in rural adult chinese populations of the Bai nationality in Dali: the Yunnan Minority Eye Study. *Investigative ophthalmology & visual science* **53**, 6617, doi:10.1167/iovs.11-8947 (2012).

13 Zhong, H. *et al.* Ethnic Variations in Pterygium in a Rural Population in Southwestern China: The Yunnan Minority Eye Studies. *Ophthalmic Epidemiol* **23**, 116-121, doi:10.3109/09286586.2015.1099685 (2016).

14 Asokan, R., Venkatasubbu, R. S., Velumuri, L., Lingam, V. & George, R. Prevalence and associated factors for pterygium and pinguecula in a South Indian population. *Ophthalmic Physiol Opt* **32**, 39-44, doi:10.1111/j.1475-1313.2011.00882.x (2012).

15 Nangia, V. *et al.* Prevalence and associated factors for pterygium in rural agrarian central India. The central India eye and medical study. *PLoS One* **8**, e82439, doi:10.1371/journal.pone.0082439 (2013).

16 Marmamula, S., Khanna, R. C. & Rao, G. N. Population-based assessment of prevalence and risk factors for pterygium in the South Indian state of Andhra Pradesh: the Andhra Pradesh Eye Disease Study. *Invest Ophthalmol Vis Sci* **54**, 5359-5366, doi:10.1167/iovs.13-12529 (2013).

17 Gazzard, G. *et al.* Pterygium in Indonesia: prevalence, severity and risk factors. *Br J Ophthalmol* **86**, 1341-1346, doi:10.1136/bjo.86.12.1341 (2002).

18 Fotouhi, A., Hashemi, H., Khabazkhoob, M. & Mohammad, K. Prevalence and risk factors of pterygium and pinguecula: the Tehran Eye Study. *Eye (Lond)* **23**, 1125-1129, doi:10.1038/eye.2008.200 (2009).

19 Rezvan, F. *et al.* The prevalence and determinants of pterygium and pinguecula in an urban population in Shahroud, Iran. *Acta medica Iranica* **50**, 689-696 (2012).

20 Nemet, A. Y., Vinker, S., Segal, O., Mimouni, M. & Kaiserman, I. Epidemiology and Associated Morbidity of Pterygium: A Large, Community-Based Case-Control Study. *Semin Ophthalmol* **31**, 446-451, doi:10.3109/08820538.2014.962169 (2016).

21 Tano, T. *et al.* Prevalence of pterygium in a population in Northern Japan: the Locomotive Syndrome and Health Outcome in Aizu Cohort Study. *Acta Ophthalmologica* **91**, e232-e236, doi:10.1111/aos.12044 (2013).

22 Lim, C. Y., Kim, S. H., Chuck, R. S., Lee, J. K. & Park, C. Y. Risk Factors for Pterygium in Korea: The Korean National Health and Nutrition Examination Survey V, 2010-2012. *Medicine (Baltimore)* **94**, e1258, doi:10.1097/MD.0000000000001258 (2015).

23 Pyo, E. Y., Mun, G. H. & Yoon, K. C. The prevalence and risk factors for pterygium in South Korea: the Korea National Health and Nutrition Examination Survey (KNHANES) 2009-2010. *Epidemiol Health* **38**, e2016015, doi:10.4178/epih.e2016015 (2016).

24 Rim, T. H., Nam, J., Kim, E. K. & Kim, T. I. Risk factors associated with pterygium and its subtypes in Korea: the Korean National Health and Nutrition Examination Survey 2008-2010. *Cornea* **32**, 962-970, doi:10.1097/ICO.0b013e3182801668 (2013).

25 Durkin, S. R. *et al.* The prevalence, severity and risk factors for pterygium in central Myanmar: the Meiktila Eye Study. *Br J Ophthalmol* **92**, 25-29, doi:10.1136/bjo.2007.119842 (2008).

26 Bikbov, M. M. *et al.* Pterygium Prevalence and Its Associations in a Russian Population: The Ural Eye and Medical Study. *Am J Ophthalmol* **205**, 27-34, doi:10.1016/j.ajo.2019.02.031 (2019).

27 Ang, M. *et al.* Prevalence of and racial differences in pterygium: a multiethnic population study in Asians. *Ophthalmology* **119**, 1509-1515, doi:10.1016/j.ophtha.2012.02.009 (2012).

28 Cajucom-Uy, H., Tong, L., Wong, T. Y., Tay, W. T. & Saw, S. M. The prevalence of and risk factors for pterygium in an urban Malay population: the Singapore Malay Eye Study (SiMES). *Br J Ophthalmol* **94**, 977-981, doi:10.1136/bjo.2008.150847 (2010).

29 Wong, T. Y., Foster, P. J., Johnson, G. J., Seah, S. K. & Tan, D. T. The prevalence and risk factors for pterygium in an adult Chinese population in Singapore: the Tanjong Pagar survey. *Am J Ophthalmol* **131**, 176-183, doi:10.1016/s0002-9394(00)00703-0 (2001).

30 West, S. & Munoz, B. Prevalence of pterygium in Latinos: Proyecto VER. *Br J Ophthalmol* **93**, 1287-1290, doi:10.1136/bjo.2008.152694 (2009).

31 McCarty, C. A., Fu, C. L. & Taylor, H. R. Epidemiology of pterygium in Victoria, Australia. *Br J Ophthalmol* **84**, 289-292, doi:10.1136/bjo.84.3.289 (2000).
